# One-Step Microwave Synthesis of Micro/Nanoscale LiFePO_4_/Graphene Cathode With High Performance for Lithium-Ion Batteries

**DOI:** 10.3389/fchem.2020.00104

**Published:** 2020-02-25

**Authors:** Shulong Liu, Ping Yan, Haibin Li, Xiaobo Zhang, Wei Sun

**Affiliations:** ^1^School of Physics and Electronic Information/Information College, Huaibei Normal University, Huaibei, China; ^2^School of Life Science, Huaibei Normal University, Huaibei, China

**Keywords:** LiFePO_4_/graphene composite, micro/nanoscale, one step, microwave synthesis, electrochemical performance

## Abstract

In this study, micro/nanoscale LiFePO_4_/graphene composites are synthesized successfully using a one-step microwave heating method. One-step microwave heating can simplify the reduction step of graphene oxide and provide a convenient, economical, and effective method of preparing graphene composites. The structural analysis shows that LiFePO_4_/graphene has high phase purity and crystallinity. The morphological analysis shows that LiFePO_4_/graphene microspheres and micron blocks are composed of densely aggregated nanoparticles; the nanoparticle size can shorten the diffusion path of lithium ions and thus increase the lithium-ion diffusion rate. Additionally, the graphene sheets can provide a rapid transport path for electrons, thus increasing the electronic conductivity of the material. Furthermore, the nanoparticles being packed into the micron graphene sheets can ensure stability in the electrolyte during charging and discharging. Raman analysis reveals that the graphene has a high degree of graphitization. Electrochemical analysis shows that the LiFePO_4_/graphene has an excellent capacity, high rate performance, and cycle stability. The discharge capacities are 166.3, 156.1, 143.0, 132.4, and 120.9 mAh g^−1^ at rates of 0.1, 1, 3, 5, and 10 C, respectively. The superior electrochemical performance can be ascribed to the synergy of the shorter lithium-ion diffusion path achieved by LiFePO_4_ nanoparticles and the conductive networks of graphene.

## Introduction

Energy and materials, important pillars of the modern developing society, are closely related to human civilization. Rechargeable lithium-ion batteries, an environmentally friendly and new green energy, have wide applicability in the fields of energy storage and transportation (Song et al., 2018). The ever-increasing demand for high current charge-discharge capability, high energy density, and long service life has driven the development of the lithium battery industry (Zhou et al., 2019). Olivine phase lithium iron phosphate (LiFePO_4_) is one of the focused cathode materials in lithium-ion batteries (Padhi et al., 1997a,b). It has many superior properties, such as that Fe is low-cost and environmentally benign, that the covalently bonded PO_4_ groups make the chemical properties more stable and prolong service lifetime, and that it has a high theoretical capacity (170 mAhg^−1^) and flat voltage plateau (3.45 V vs. Li/Li^+^). However, LiFePO_4_ materials have some drawbacks, such as inferior electronic conductivity (ca.10^−9^–10^−10^ Scm^−1^) as well as slow one-dimensional lithium ion diffusion, which is a formidable obstacle to the high performance of lithium-ion batteries (Goodenough and Kim, 2010; Dathar et al., 2011). A considerable number of methods have been adopted with the aim of alleviating the above shortcomings. These methods can be categorized into two main classes: particle size control (Prosini et al., 2003; Zhao et al., 2016) and conductive material coating (Chang et al., 2019; Han et al., 2019; Ma et al., 2019; Tao et al., 2019).

Small particle size can decrease the migration distance of lithium ions from the interior to the surface and increase the diffusion rate (Lim et al., 2008; Hai et al., 2019; Li et al., 2019; Xiao et al., 2019). Various techniques, including solid-state reaction (Zheng et al., 2008), sol-gel (Zhang et al., 2011) hydrothermal (Kiyoshi et al., 2008; Chang et al., 2014), co-precipitation (Park et al., 2003; Wang et al., 2013), and microwave heating (Wang et al., 2007; Beninati et al., 2008; Guo et al., 2010), are adopted to control particle size. Moreover, surface coating with conductive material can increase the electronic conductivity between particles (Wang et al., 2010; Fathollahi et al., 2015; Ahn et al., 2019) and provide paths in all directions for the fast transmission of electrons (Wang et al., 2009; Jang et al., 2011; Fan et al., 2014). Graphene with high electrical conductivity has been adopted to improve the cycling stability and rate capability of cathode material (Ding et al., 2010; Zhou et al., 2011; Shi et al., 2012; Tang et al., 2012; Chen et al., 2018; Wang et al., 2018). Ding et al. (2010) prepared nano-structured LiFePO_4_/graphene using co-precipitation and sintering at 700°C for 18 h under argon flow. Shi et al. (2012) prepared graphene-wrapped LiFePO_4_/C using a microwave-assisted hydrothermal method, followed by sintering at 600°C for 2 h under H_2_/Ar flow. Zhou et al. (2011) first synthesized LiFePO_4_ nanoparticles by a hydrothermal method and then synthesized LiFePO_4_/graphene from LiFePO_4_ nanoparticles and graphene oxide nanosheets by spray-drying and annealing processes. Tang et al. (2012) synthesized LiFePO_4_/graphene by mixing three-dimensional graphene prepared by chemical vapor deposition and LiFePO_4_ prepared by solid-state reaction in a N-methyl pyrrolidinone (NMP) suspension. The above experimental methods are very complicated, and most of them require long-term high-temperature treatment and atmosphere protection, which lead to high energy consumption and cost. Additionally, the graphene and active materials agglomerate easily and distribute unevenly. Therefore, simplifying the preparation technology and obtaining a product with a small and homogeneous distribution remain great challenges for preparing LiFePO_4_/graphene composites. Microwave heating is a convenient, economical, and environmentally friendly route for the preparation of graphene composites in a way that addresses the deficiency of graphene modification. Microwave heating can simplify the reduction step of graphene oxide, as, due to the microwave-absorbing properties of graphene oxide, microwave irradiation can restore it into graphene directly without any reductive agent and atmosphere.

In this work, micro/nanoscale LiFePO_4_/graphene composites are synthesized successfully using a one-step microwave heating method. The synthesis technique has a decisive influence on the structure, morphology, and electrochemical properties of the LiFePO_4_ product. Microwave synthesis can save synthesis time; this is because the raw material can absorb microwave energy by itself and convert electromagnetic energy into heat and internal molecular kinetic energy, thus improving the diffusion coefficient and accelerating the reaction rate. Meanwhile, microwave synthesis can lower the synthesis temperature; this is because the electromagnetic field decreases the activation energy of the reaction. Therefore, microwave heating is a rapid and effective synthetic method for preparing a product with small particle size. Furthermore, unlike in complex, multi-step preparation processes, microwave irradiation can restore the graphene oxide into graphene directly without any reductive agent and atmosphere. The synthesized micro/nanoscale LiFePO_4_/graphene composites with fine particle size and uniform distribution can decrease the migration distance of lithium ions from the interior to the surface and increase the diffusion rate. Meanwhile, graphene wrapping of the surface of LiFePO_4_ particles can guarantee that the electrons migrate to the active sites quickly. Controlling the particle size and coating with graphene play important roles in the electrochemical performance. The effects of graphene and microwave irradiation on the electrochemical performance of LiFePO_4_/graphene cathode materials for lithium-ion batteries are further investigated.

## Experimental

### Preparation of Materials

FeSO_4_·7H_2_O (99%), LiOH·H_2_O (95%), H_3_PO_4_ (85%), ethylene glycol, and sucrose were purchased from Sinopharm Chemical Reagent Co. Ltd. Graphene oxide was synthesized from natural graphite powder (325 mesh) using a modified Hummers' method (Kovtyukhova et al., 1999; Stankovich et al., 2007).

The LiFePO_4_/graphene and LiFePO_4_/C composites were synthesized via the following steps. FeSO_4_·7H_2_O and H_3_PO_4_ were dissolved in a mixed solution of de-ionized water and ethylene glycol, and GO suspension was added to the solution. Next, a mixture of LiOH·H_2_O aqueous solution and GO suspension was added into the mixed solution under constant stirring. The molar ratio of Li:Fe:P is 3:1:1. After stirring for 3 h, the solution was evaporated at 80°C for 12 h. Meanwhile, a separate sample was prepared with the GO suspension replaced by sucrose as the source of carbon, and the previous steps were repeated. Finally, the precursors obtained were pressed into pellets, and then the pellets were placed inside a quartz crucible with a cover to prevent air oxidation. The quartz crucible was put in the middle of the domestic microwave oven, and the precursors were radiated by microwave for 10 min with a maximum power of 1,500 W and a frequency of 2.45 GHz. After microwave irradiation, LiFePO_4_/graphene and LiFePO_4_/C composites were obtained, respectively.

### Characterization Techniques

The structures of LiFePO_4_/graphene and LiFePO_4_/C composites were investigated using an X-ray diffractometer (X'pert PRO, Panalytical, Holland) with Cu Kα radiation operated at 40 kV and 40 mA. The contents of graphene and carbon in the LiFePO_4_/graphene and LiFePO_4_/C composites were calculated from TG-DSC (STA449F3, NETZSCH, Germany), which was carried out from room temperature to 700°C under an air atmosphere at a rate of 10°C min^−1^. The morphologies of LiFePO_4_/graphene composites were observed using a scanning electron microscope (SEM, JSM-IT300 at 20 kV) and transmission electron microscopy (TEM, JEM2100F Japan at 200 kV). The Raman spectra of LiFePO_4_/graphene and LiFePO_4_/C composites were recorded from 100 to 3,200 cm^−1^ on a Renishaw Raman microprobe (INVIA, China) using a 514.5 nm argon-ion laser at room temperature.

### Cell Fabrication and Electrochemical Measurement

The electrochemical behaviors of the LiFePO_4_/graphene and LiFePO_4_/C composites were evaluated with 2,025 coin-type batteries. The cathode electrodes were prepared by mixing 80 wt% active materials (LiFePO_4_/graphene or LiFePO_4_/C) and 10 wt% carbon black (TIMCAL) with 10 wt% polytetrafluoroethylene (PTFE, Aldrich) in isopropyl alcohol solution (99.5%, Aldrich). A uniform slurry was formed and pasted onto Al foils, dried at 120°C for 12 h, and then cut into circular electrodes with a diameter of 10 mm. Lithium metal (99.9%, Alfa-Aesar) was used as the anode, Celgard polypropylene (Celgard 2400) as the separator, and 1M LiPF_6_ dissolved in ethylene carbonate and dimethyl carbonate (with a 1:1 volume ratio) as the electrolyte (MERCK KGaA, Germany). The cells were assembled in an argon-filled glove box (Etelux Lab2000, China). Cells were charged and discharged at room temperature using a LAND-CT2001A battery cycler (Wuhan, China) within the voltage range of 2.7–4.2 V (vs. Li^+^/Li). Cyclic voltammetry (CV) was performed with an Auto Potentiostat 30 system at a scan rate of 0.1 mVs^−1^ between 2.5 and 4.2 V. Electrochemical impedance spectroscopy (EIS) profiles were obtained at the same open-circuit voltage by applying a 5-mV amplitude of the AC voltage with the frequency ranging from 100 kHz to 0.01 Hz.

## Results and Discussion

### Phase Structural Analysis

**Figure 1:**
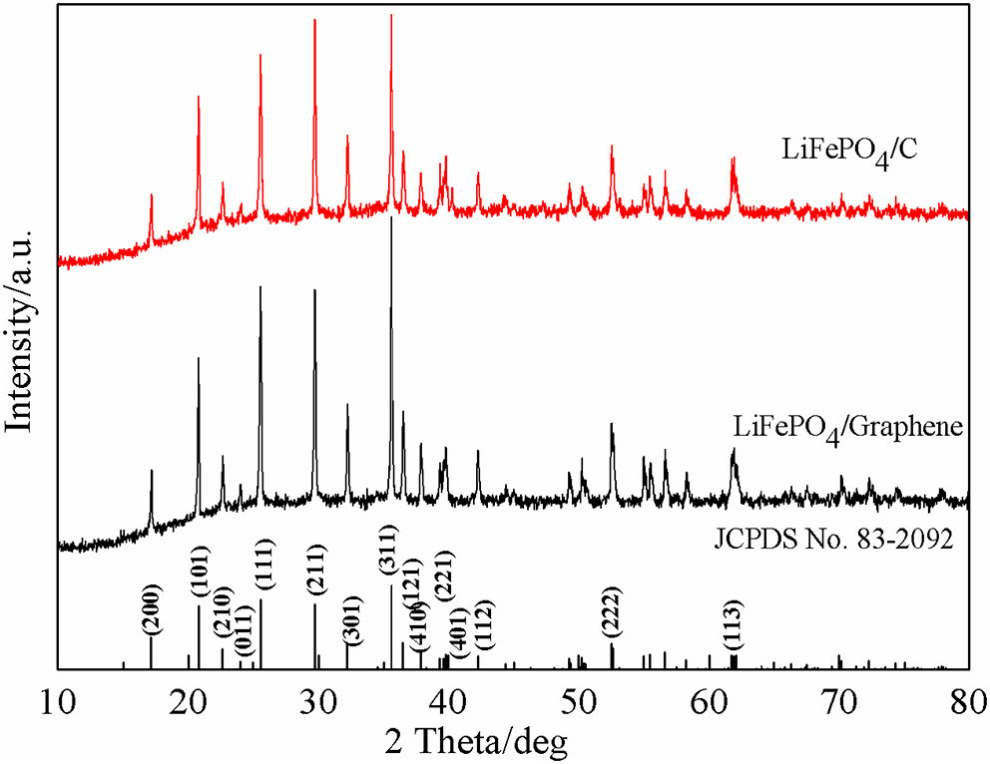
XRD patterns of the LiFePO_4_/graphene and LiFePO_4_/C composites.

The phase constitution and crystal structure of the synthetic LiFePO_4_/graphene and LiFePO_4_/C composites are here investigated. XRD patterns of the composites are shown in Figure 1. It can be seen that there is no noticeable difference between LiFePO_4_/graphene and LiFePO_4_/C composites. All the sharp diffraction peaks corresponding to the (200), (101), (210), (011), (111), (211), (301), (311), (121), (410), (221), (401), (112), (222), and (123) planes can be indexed to the orthorhombic olivine-type structure LiFePO_4_ with the Pnma space group (JCPDS card No. 83-2092) (Wang et al., 2009, 2010), and no excess impurity peaks are observed. The results manifest that the synthetic composites have high crystallinity and purity; this is mainly because microwave synthesis has the advantage of increasing the crystallinity and purity of products. The diffraction pattern of LiFePO_4_/graphene shows that no diffraction peak of graphene oxide (at around 12°) is observed, proving that the graphene oxide has already been reduced into graphene directly without any reducing agent or atmosphere. This is mainly because the graphene oxide with a large amount of oxygen functional groups on the surface that can absorb microwaves easily, and electromagnetic energy is converted into heat and molecular kinetic energy; the reactive oxygen groups are then exfoliated, and, finally, the graphene oxide is restored into graphene. Also, the introduction of graphene has no effect on the structure of LiFePO_4_. Moreover, the diffraction pattern of LiFePO_4_/C shows no diffraction peaks corresponding to residual carbon, indicating that the carbon decomposed from sucrose in the sample exists in an amorphous state.

### TG-DSC Analysis

**Figure 2:**
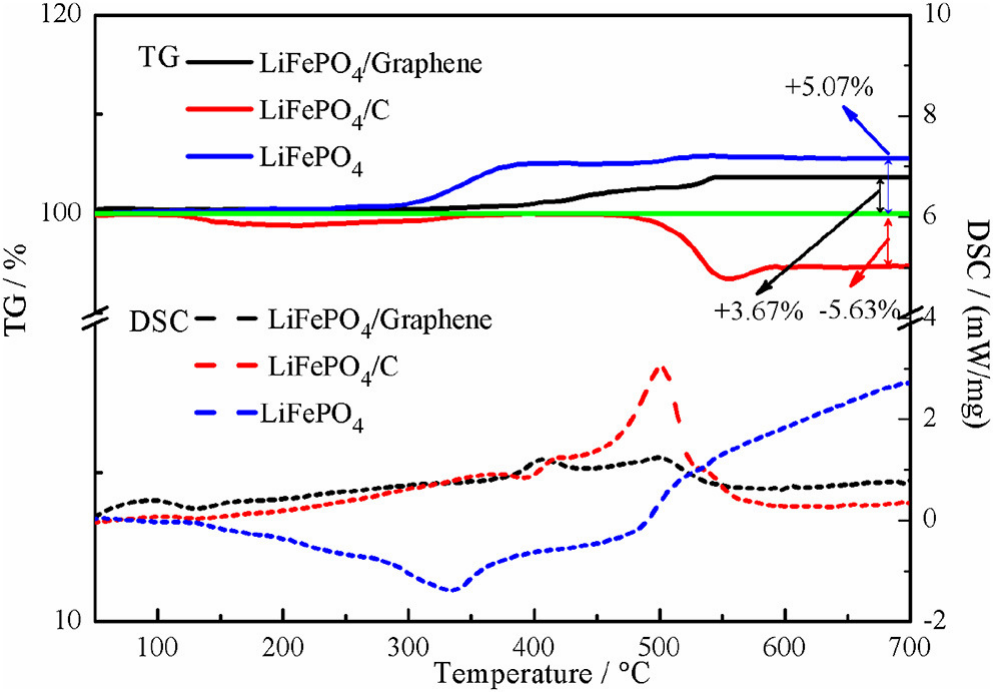
TG-DSC curves of LiFePO_4_/graphene, LiFePO_4_/C composites, and LiFePO_4_.

TG-DSC measurement data is used to estimate the graphene and carbon content in the LiFePO_4_/graphene and LiFePO_4_/carbon composites, as shown in Figure 2. The pure LiFePO_4_ can be completely oxidized to Li_3_Fe_2_(PO_4_)_3_ and Fe_2_O_3_ under air flow, and the total weight gain is about 5.07% in theory (Belharouak et al., 2005; Bai et al., 2015). For LiFePO_4_/graphene and LiFePO_4_/carbon composites, in the temperature range of 400–600°C, the graphene and carbon are oxidized to CO_2_ gas, so the amounts of graphene and carbon in the LiFePO_4_/graphene and LiFePO_4_/carbon composites are about 1.40 and 10.70%, respectively.

### Raman Analysis

**Figure 3:**
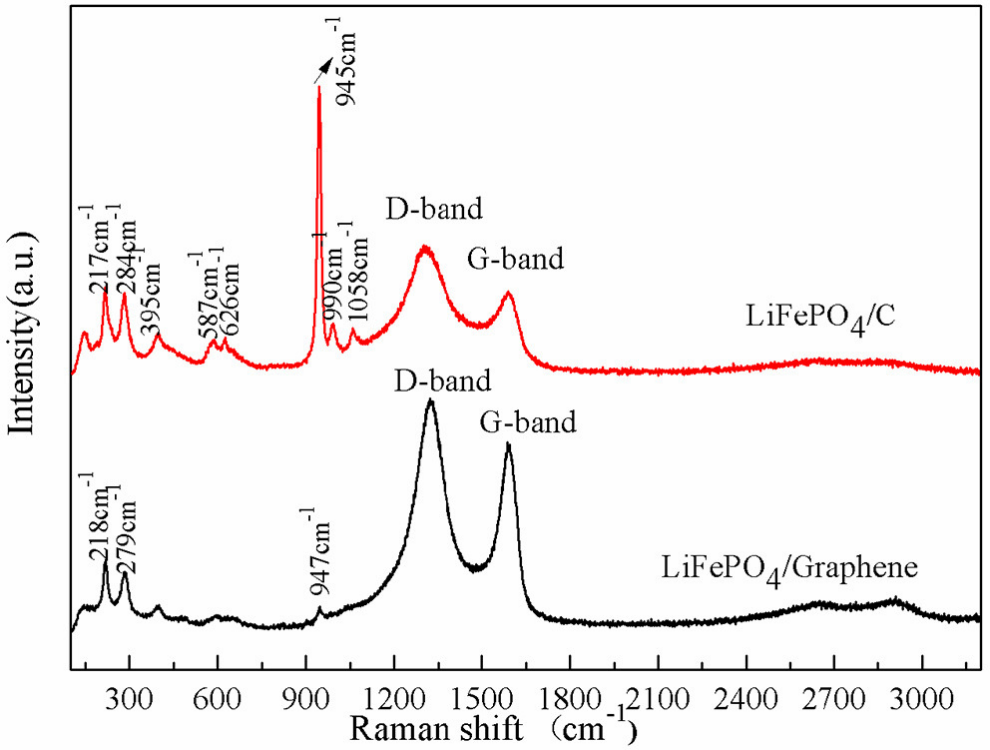
Raman spectra of LiFePO_4_/graphene and LiFePO_4_/C composites.

Raman scattering spectroscopy was employed to recognize the chemical structure of the LiFePO_4_/graphene and LiFePO_4_/C composites; the results are shown in Figure 3. The main vibration modes include internal modes attributable to ${\text{PO}}_{4}^{3-}$ anions and external modes attributable to the coupled motion of Fe^2+^ and ${\text{PO}}_{4}^{3-}$ (Markevich et al., 2011). The modes at 990, 1,058, and 945 cm^−1^ correspond to the anti-symmetric (ν3) and symmetric (ν1) stretching of the P–O bonds. The modes at 626 and 587 cm^−1^ correspond to the symmetric (ν2) and anti-symmetric (ν4) bending of the O-P-O angles. The mode at 395 cm^−1^ corresponds to the lithium cage and oxygen ion breathing cage. The modes in the 100–300 cm^−1^ range are induced by translation of Fe and coupled translation and vibration of Fe and ${\text{PO}}_{4}^{3-}$ (Burba and Frech, 2004; Wu et al., 2013). Moreover, there are two obvious D band peaks at around 1,310 cm^−1^ and a G band at around 1,590 cm^−1^ (Tuinstra and Koenig, 1970). The D band is induced by a disordered and defective carbon structure in the crystal plane of the short-order *sp*^2^ and *sp*^3^ carbon. The G band is assigned to the in-plane bond-stretching motion of *sp*^2^ carbon atoms. The intensity ratio of the D and G bands (*I*_D_/*I*_G_) is inversely proportional to the degree of graphitization of carbon materials. The *I*_D_/*I*_G_ in LiFePO_4_/graphene composites is 1.18, while the *I*_D_/*I*_G_ in LiFePO_4_/C composites is 1.43. This implies that the graphene has a higher degree of graphitization than the carbon decomposed from sucrose. The higher the degree of graphitization, the better the conductivity of the carbon. A high degree of graphitization is favorable for electron transfer and improves the electrochemical performance of the cathode. Additionally, the strong signals of the graphene (D band and G band) weaken and override the bands of LiFePO_4_ in the high-frequency region.

### Morphological Analysis

**Figure 4:**
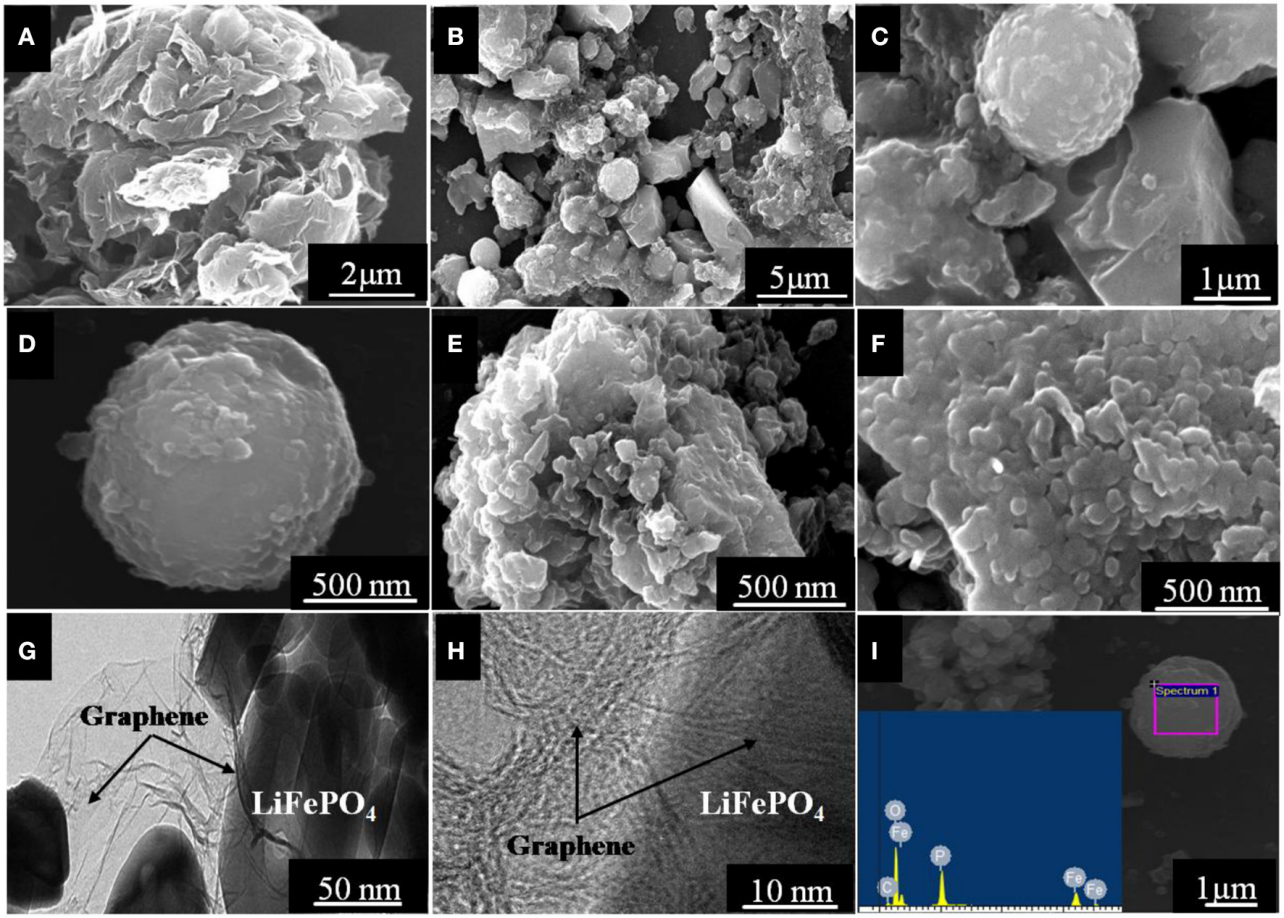
SEM images of the graphene oxide **(A)** and LiFePO_4_/graphene **(B–F)**; TEM and HRTEM images of the LiFePO_4_/graphene composites **(G,H)**; EDS spectra of the LiFePO_4_/graphene composites **(I)**.

SEM images of the graphene oxide and LiFePO_4_/graphene are shown in Figures 4A–F. Figure 4A shows that the micron-scale graphene oxide sheets aggregate into petal shapes; these sheets can provide implantation sites for the adhesion of reaction particles. Figures 4B,C clearly shows that the LiFePO_4_/graphene composites are composed of micron-scale spheres and blocks with average dimensions of ~2 μm. In Figures 4D–F, it can be clearly observed that these LiFePO_4_/graphene microspheres and micron blocks are composed of densely aggregated nanoparticles. This structure forms because the self-heating effect induced by the microwave heating can greatly shorten the reaction time, and the graphene wrapping the surface of LiFePO_4_ particles can inhibit the growth of grains. Under the action of graphene, the nanoparticles assembled into microspheres and micron blocks. When the highly conductive electrolyte penetrates into the cathode material, the nanoparticles have a high specific surface area, which increases the contact area with the electrolyte. The nanoparticle size can shorten the diffusion paths of electrons and lithium ions and improve the conductivity of the cathode material significantly. Moreover, the micron structure formed by the aggregation of nanoparticles does not collapse during the process of lithium-ion intercalation and deintercalation, which ensures the stability of cathode material in the electrolyte. TEM and HRTEM images of the micron/nanoscale LiFePO_4_/graphene composite are shown in Figures 4G,H. The ultrathin graphene sheets successfully form an effective conducting network and intrinsically bridge and intimately connect the active LiFePO_4_ particles. Figure 4H indicates that the graphene sheets around LiFePO_4_ are highly graphitic. The highly efficient and stable conducting network can give the material desirable electrochemical properties. In the energy spectrum, elements of P, O, Fe, and C are found, as shown in Figure 4I; Li cannot be detected because of its very low atomic weight.

**Figure 5:**
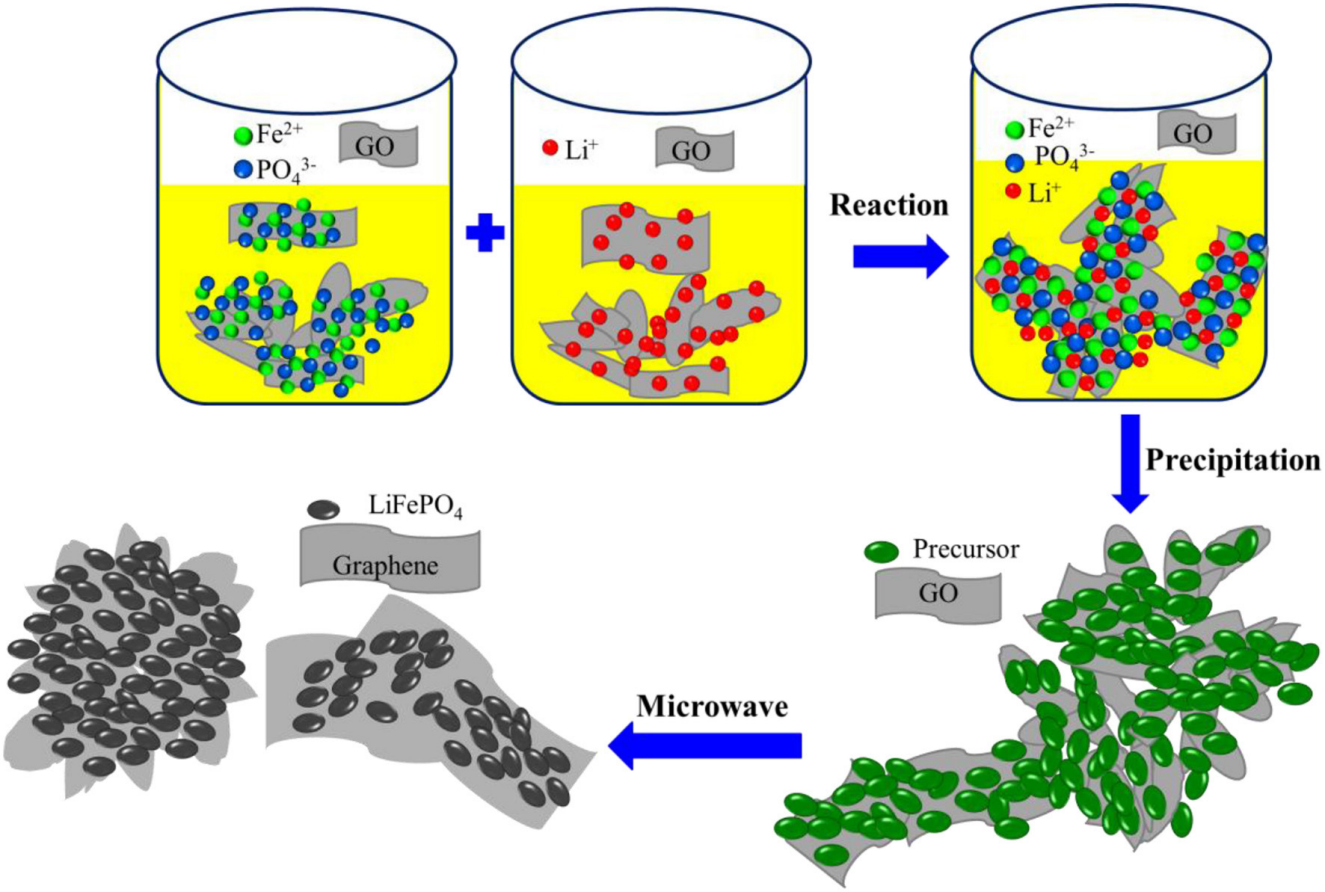
Schematic illustration of the formation process of LiFePO_4_/graphene composites.

The formation process of the LiFePO_4_/graphene composites is illustrated in Figure 5. At the initial stage of the reaction, the chemical reaction follows a dissolution–precipitation mechanism. The iron ions, phosphate ions, and lithium ions in the solution react with each other and form agglomerated precipitate on the surface of the graphene oxide sheets, and a large number of active functional groups are adsorbed on the surface of graphene oxide. At the stage of microwave irradiation, the active functional groups, being polar molecules, can absorb microwave easily, and electromagnetic energy is converted into heat and molecular kinetic energy. The temperature increase quickly, the reactive oxygen groups are exfoliated, and, finally, the graphene oxide is restored into graphene. Meanwhile, the precipitated particles adsorbed on the surface of reduced graphene sheets become hot and absorb microwaves quickly, the particles interact with each other, and then crystal nuclei are formed quickly under the action of the microwave electromagnetic field. Finally, under the influence of micron graphene sheets, the crystal nuclei grow, agglomerate, and form microspheres and micron blocks.

### Electrochemical Properties Analysis

**Figure 6:**
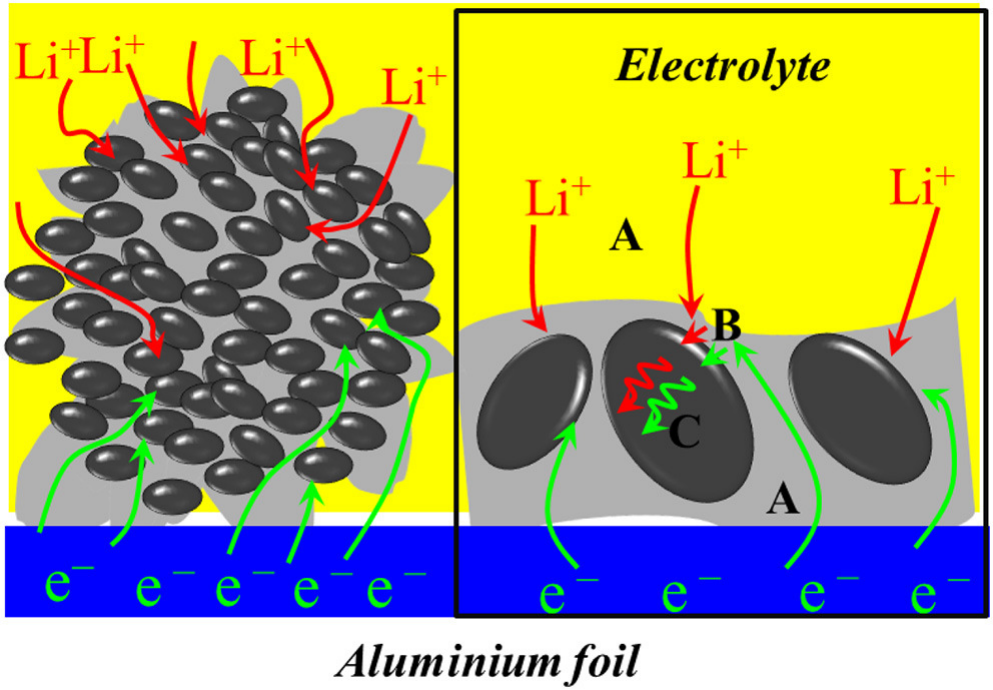
Schematic diagram of LiFePO_4_/graphene electrode dynamics.

A schematic diagram of LiFePO_4_/graphene electrode dynamics is shown in Figure 6. Transportation of electrons and ions (e^−^ and Li^+^) from their “reservoirs” toward the LiFePO_4_ particles (Gaberscek et al., 2007; Gaberscek, 2009) is shown as step A. A charge incorporation reaction that involves the transfer of e^−^ and Li^+^ from the outside into the interior of active particles is shown as step B, and the transport of the lithium component inside the solid active particles (solid-state diffusion) is shown as step C. It can be seen that graphene can provide a high-speed channel for the rapid diffusion of electrons and cause the electrons to reach the reactive site quickly, thus increasing the electronic conductivity of the materials. Meanwhile, the nanoparticles can shorten the transport path of Li^+^ from the surface to the interior of solid active particles and improve the diffusion coefficient of lithium ions. Moreover, the nanoparticles are surrounded by the micron graphene sheets, and the micron structure guards the stability of the material. Therefore, LiFePO_4_/graphene composites are expected to have excellent electrochemical performance.

**Figure 7:**
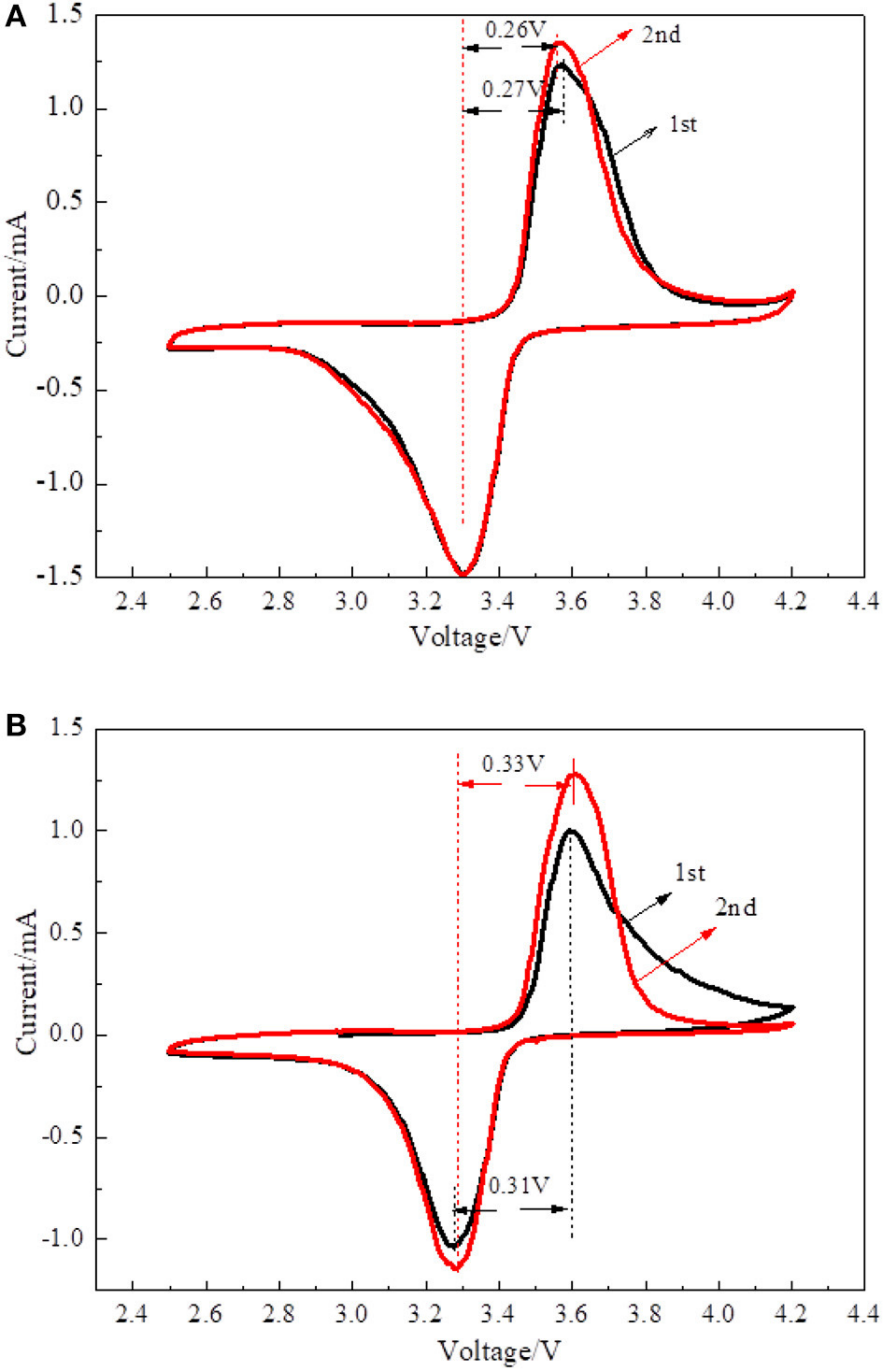
Cyclic voltammetry curves of LiFePO_4_/graphene **(A)** and LiFePO_4_/C **(B)** composites.

Cyclic voltammetry was performed to investigate the electrochemical kinetics of LiFePO_4_/graphene and LiFePO_4_/C cathode materials. Figure 7 shows the CV spectra of the LiFePO_4_/graphene and LiFePO_4_/C composites. In the first scan, there is a pair of redox peaks corresponding to the Fe^2+^/Fe^3+^ couple (Ding et al., 2010; Zhou et al., 2011). The shapes of redox peaks are low and asymmetrical; this is because, in the first charging and discharging cycle, active materials are not completely saturated by electrolyte, and the pathways of lithium ion insertion and extraction were not completely formed. In the second scan, the current intensity increases, and the shape of the redox peaks becomes more symmetrical and sharper. For LiFePO_4_/graphene, the potential difference between the oxidation and reduction peaks decreases from 0.27 to 0.26 V, which means that the phase is stabilized in subsequent cycles. Figure 7B shows the CV spectra of LiFePO_4_/C composites. During the second scan, the potential difference increases from 0.31 to 0.33 V, which proves that detrimental polarization becomes more and more serious. The results show that LiFePO_4_/graphene composites have very high reversibility and better electrochemical activity.

**Figure 8:**
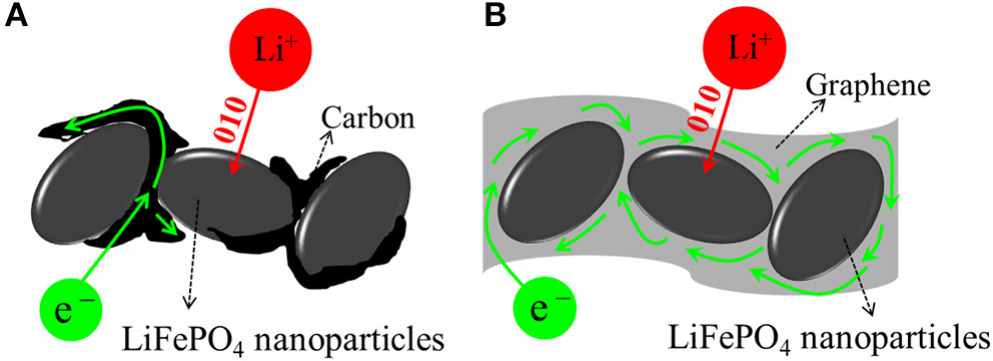
Electron transfer pathway in LiFePO_4_/graphene **(A)** and LiFePO_4_/C **(B)** electrodes.

The polarization of the LiFePO_4_/C electrode is explained by the electron transfer pathway, as shown in Figure 8A. The carbon is dispersed unevenly, so the electrons cannot reach the entire reactive site where the Li^+^ ions intercalate. In contrast, for LiFePO_4_/graphene, due to the one-dimensional Li^+^ ion mobility in the framework, the graphene can ensure that electrons reach particles from all directions and alleviate the polarization, as shown in Figure 8B. Therefore, the LiFePO_4_/graphene composites, with well-defined peaks and smaller potential difference, have higher electrochemical reactivity.

**Figure 9:**
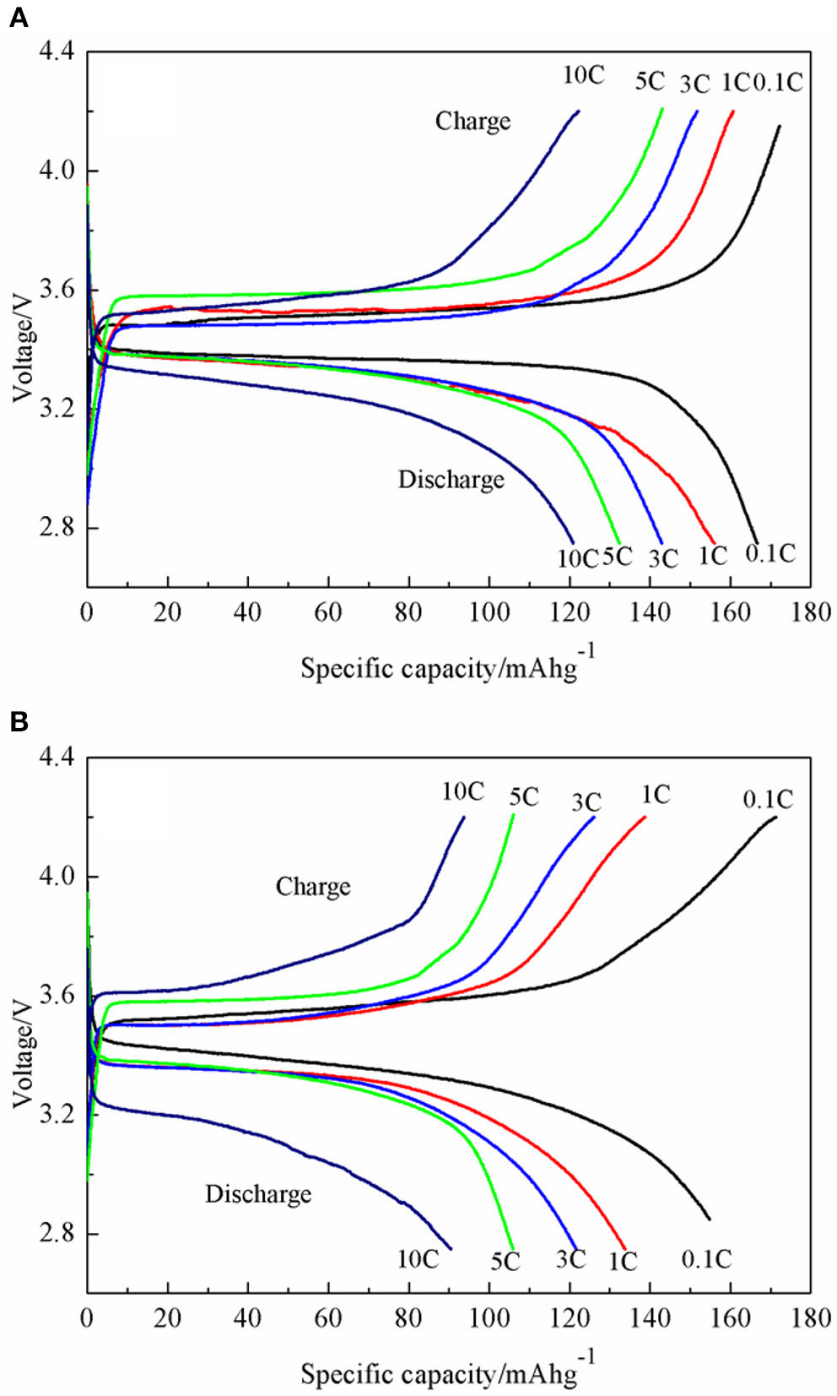
Charge/discharge profiles of LiFePO_4_/graphene **(A)** and LiFePO_4_/C **(B)** composites at various current rates ranging from 0.1 to 10 C.

**Figure 10:**
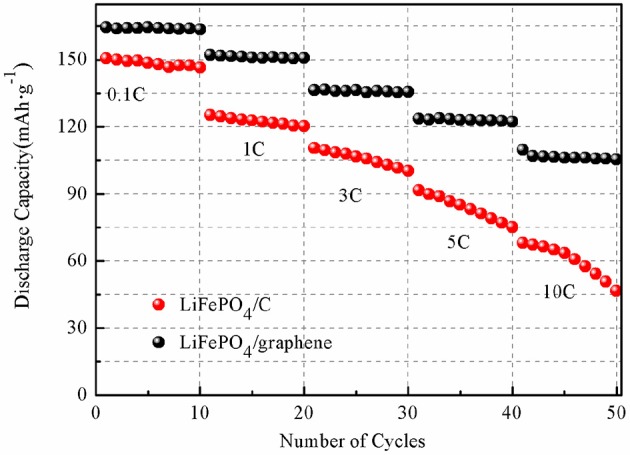
Cycling performances of LiFePO_4_/graphene **(A)** and LiFePO_4_/C **(B)** composites at various current rates ranging from 0.1 to 10 C.

The charging and discharging capacity profiles of the LiFePO_4_/graphene and LiFePO_4_/C at progressively increasing C rates from 0.1 to 10 C are shown in Figure 9. The cells are cycled in the voltage window of 2.7–4.2 V at room temperature. For LiFePO_4_/graphene composites, the initial discharge capacity is 166.3 mAhg^−1^ at 0.1 C, and the discharge capacity decreases to 156.1 mAhg^−1^ with an increase in the discharge rate to 1 C. At a higher discharge rate of 5 C, the cell delivers a capacity of 132.4 mAhg^−1^. Even at a 10 C rate, the capacities can reach 120.9 mAhg^−1^, and a good voltage plateau remains above 3 V. For LiFePO_4_/C, the discharge capacity is 154.8, 133.8, 121.6, 105.9, and 86.4 mAh g^−1^ at 0.1, 1, 3, 5, and 10 C rates, respectively. The cycling performances of the LiFePO_4_/graphene and LiFePO_4_/C from 0.1 to 10 C are shown in Figure 10. Although for LiFePO_4_/graphene, the specific capacity decreases with increasing current rate, the capacity retention remains very good for all of the different rates; the discharge capacity retentions are, respectively, 99.5, 99.2, 99.4, 99.1, and 97.1% at 0.1, 1, 3, 5, and 10 C current rates after being cycled 10 times. While for LiFePO_4_/C, the discharge capacity retentions are, respectively, 97.7, 96.9, 93.0, 87.1, and 79.5% at 0.1, 1, 3, 5, and 10 C current rates. All of the results demonstrate that LiFePO_4_/graphene composites have better rate performance and cycling stability. This can be attributed to the excellent electrical conductivity of graphene, which can improve the conductivity and stability of materials.

**Figure 11:**
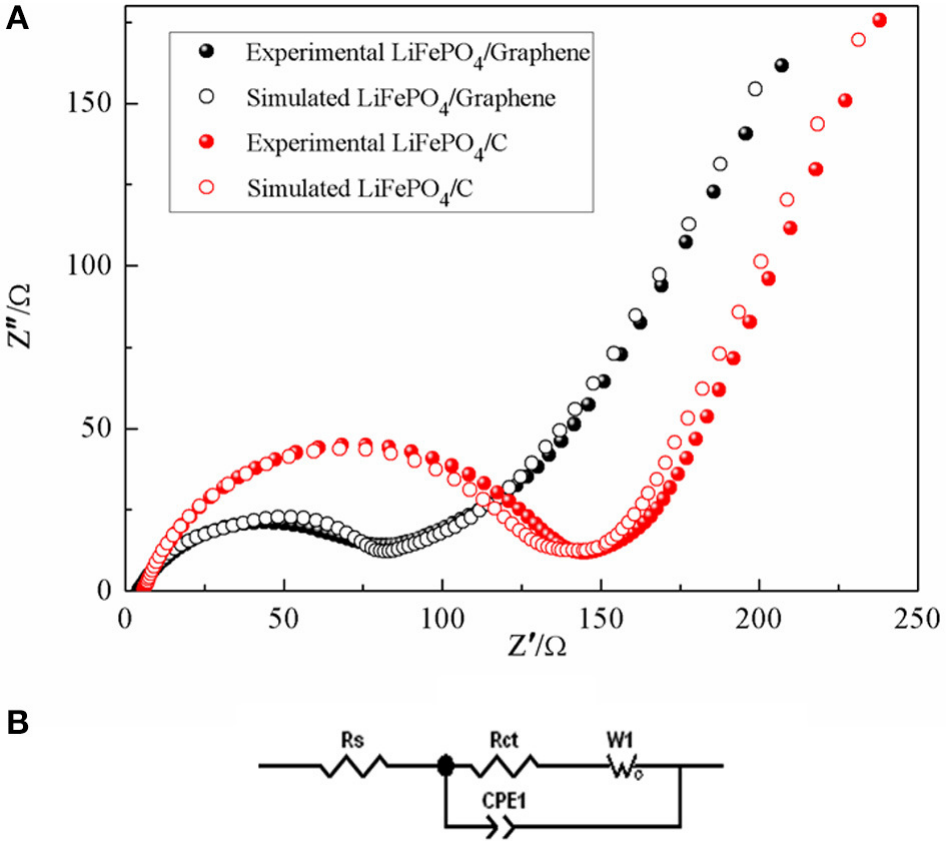
Experimental and simulated electrochemical impedance spectra of LiFePO_4_/graphene and LiFePO_4_/C composites **(A)**; equivalent circuit **(B)**.

Electrochemical impedance spectroscopy was used to investigate the electrochemical behaviors of LiFePO_4_/graphene and LiFePO_4_/C cathodes. Figure 11A shows the Nyquist plots of the LiFePO_4_/graphene and LiFePO_4_/C cathodes. The experimental EIS data is simulated by Zview2.1 software according to the equivalent circuit as shown in Figure 11B. It can be found that all the Nyquist plots present a high-frequency quasi-semicircle, which is related to the migration of the Li^+^ ions at the electrode/electrolyte interface and the charge transfer process. Meanwhile, a low-frequency sloping line is related to the Warburg impedance of the lithium-ion diffusion in the electrode (Zhang et al., 2011). *R*_S_ is the internal resistance of the cell and corresponds to the electrodes, electrolyte, and the separator resistance, *R*ct is associated with the charge-transfer resistance, *CPE* is associated with the capacitance contributed by the surface of the active material (Guo et al., 2010). The simulation results show that the *R*ct value of the LiFePO_4_/graphene cathode is 79 Ω, which is smaller than the 129 Ω value of the LiFePO_4_/C cathode. The result shows that graphene can reduce the charge transfer resistance of Li-ion insertion and extraction between the electrode/electrolyte and increase the conductivity of the LiFePO_4_/graphene cathode.

## Conclusion

A LiFePO_4_/graphene composite was successfully prepared as cathode material through one-step microwave heating. The graphene oxide, which has excellent microwave-absorbing properties, can react with microwaves quickly and be restored into high-quality graphene directly without any reducing agent or atmosphere. The introduction of graphene does not impact the structure of LiFePO_4_, and LiFePO_4_ nanoparticles are packed into micron graphene sheets. The graphene network, which has a high degree of graphitization, can provide a high-speed channel for the rapid transfer of electrons and thus increase the electronic conductivity of materials. Meanwhile, the nanoparticles can improve the diffusion coefficient of lithium ions. Moreover, because the nanoparticles are surrounded by the graphene sheets, the micron structure guards the stability of the material. The electrochemical analyses reveal that the LiFePO_4_/graphene composites have excellent high-rate performance and cycling life. The outstanding electrochemical performance, as well as the fast and efficient method, make this technology commercially viable.

## Data Availability Statement

The datasets generated for this study are available on request to the corresponding author.

## Author Contributions

SL, PY, and WS performed the experiments. SL, PY, HL, and XZ performed the data analysis. SL and PY wrote the paper. All authors contributed to the theoretical analysis and the general discussion.

### Conflict of Interest

The authors declare that the research was conducted in the absence of any commercial or financial relationships that could be construed as a potential conflict of interest.
